# Health and social care professionals’ experiences of collaborative planning—Applying the person‐centred practice framework

**DOI:** 10.1002/nop2.597

**Published:** 2020-08-13

**Authors:** Ingela Jobe, Birgitta Lindberg, Åsa Engström

**Affiliations:** ^1^ Division of Nursing Department of Health Science Luleå University of Technology Luleå Sweden

**Keywords:** collaborative planning, framework analysis, person‐centred practice, qualitative study

## Abstract

**Aim:**

To explore how person‐centred practice framework can be applied to professionals participating in collaborative planning.

**Design:**

An explorative, deductive approach.

**Method:**

Eleven professionals from health care and social care participated in the study. A deductive content analysis was performed using a framework for person‐centred practice for the analysis.

**Results:**

Practicing person‐centred care and collaborative planning is a complex process that needs to take into account system factors on both the macro‐ and the microlevel. Everyone working within the system needs to apply the same approach. Using a framework analysis offered new insights into how person‐centred care is expressed in practice during collaborative planning between the patient, and healthcare and social care professionals.

## 1 INTRODUCTION

Person‐centred care (PCC) is a concept that has become widely used (Ekman, Hedman, Swedberg, & Wallengren, 2015; McCance, McCormack, & Dewing, 2011) and is a central part of policies for delivering health care and social care. At the same time, there has been a shift from hospital‐based care to home care and the way services are delivered. This includes providing integrated health care and social care around peoples’ needs that are effectively coordinated across providers. The essence of PCC is a health system designed around individuals, families and community preferences, values and needs (World Health Organization, 2015).

A requirement for delivering PCC is collaboration between healthcare and social care professionals. The collaboration acknowledges the unique expertise of various professionals and is essential for delivering high‐quality patient care (Fox & Reeves, 2015). By focusing on PCC in practice, healthcare and social care organizations want to move from fragmented, paternalistic and disease‐oriented care to relationship‐based, collaborative and holistic care (McCance et al., 2011; Washburn & Grossman, 2017). However, many health systems are struggling with effective implementation of PCC (Santana et al., 2018). Studies have shown that barriers to the implementation of PCC are the traditional practices, structures, resources, time, skills and attitudes of professionals (Eaton, Roberts, & Turner, 2015; Moore et al., 2017; Wheat et al., 2018). Facilitators of PCC implementation include organizational factors, leadership, training, support, incentives and an enabling attitude by professionals (Moore et al., 2017; Wheat et al., 2018). Social workers have in their work for a long period focused on the individual and his or hers context. Barriers to implement PCC include an increased understanding of what it means to be with and care for a person, the relationship between social work and health care and the person that respect his or her personhood (Washburn & Grossman, 2017).

Every country tends to employ its own approach to integration of health and social services. Nevertheless, common features among these approaches are holistic care assessment, comprehensive care planning and care coordination (Wodchis, Dixon, Anderson, & Goodwin, 2015). Studies have shown collaborative person‐centred care plans are associated with improvements of physical and psychological health status, capability to self‐manage (Coulter et al., 2015) and a reduction in the length of hospital stay (Ulin, Olsson, Wolf, & Ekman, 2016). However, several studies have revealed that collaborative planning is a difficult challenge and the implementation and outcome are not always satisfactory (Jansen, Heijmans, & Rijken, 2015; Reeves et al., 2014). In Sweden, the region and the municipality collaborate and establish a collaborative plan for persons needing health care and social care. In this study, we wanted to explore how the person‐centred practice framework developed by McCormack and McCance (2016) could apply to professionals participating in collaborative planning.

## 2 BACKGROUND

**FIGURE 1:**
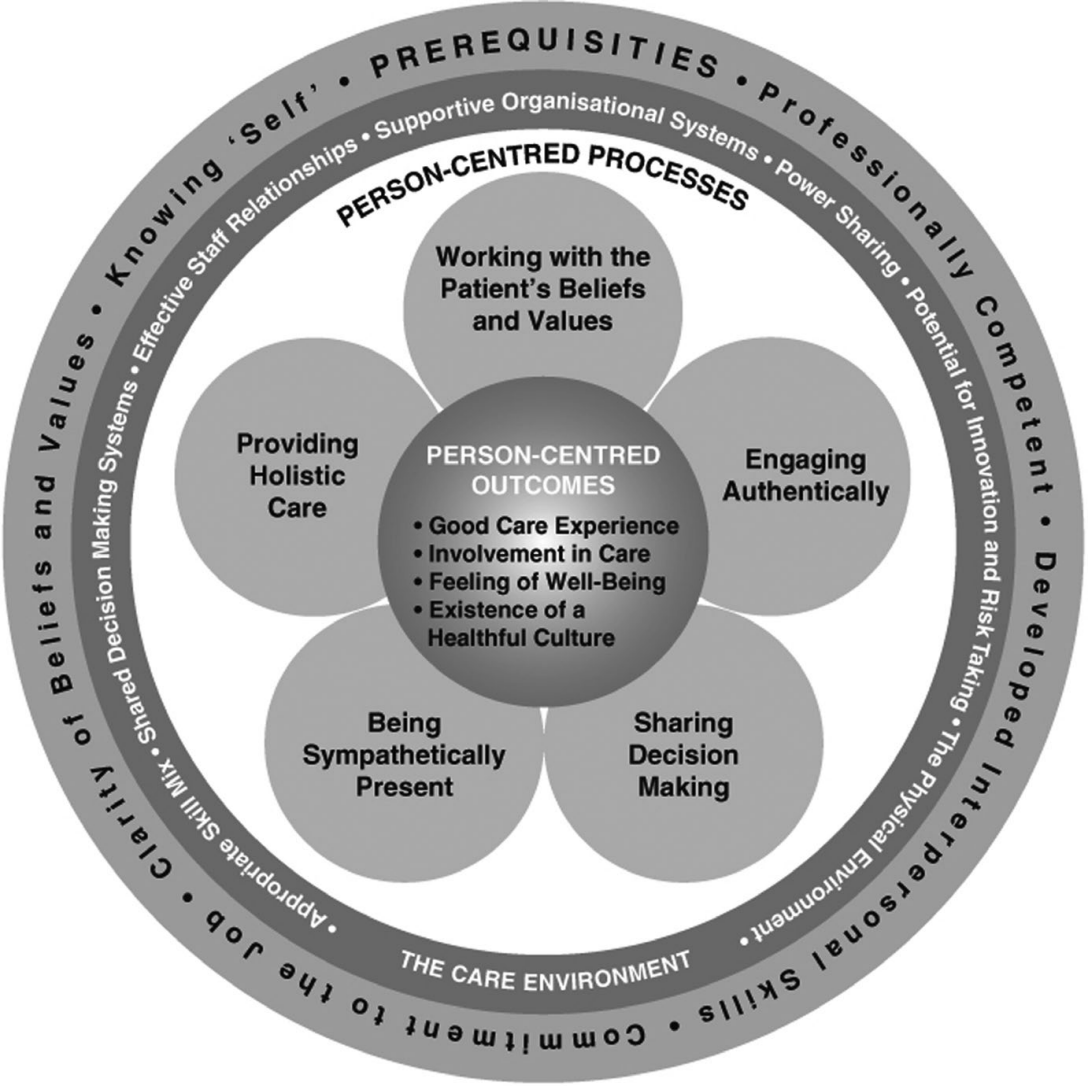
Person‐centred practice framework (McCormack & McCance, 2016)

The person‐centred practice framework developed by McCormack and McCance (2016) assists professionals and teams in understanding the elements of person‐centredness and how they can be implemented in practice. The framework has four domains: prerequisites; the care environment; person‐centred processes; and person‐centred outcomes (Figure 1). It also takes into consideration multidisciplinary and interprofessional settings and the complexity of person‐centred practice.

The prerequisites focus on the professionals, and the attributes needed to deliver PCC (McCormack & McCance, 2016). The attributes are being professionally competent, having developed interpersonal skills, being committed to the job, being able to demonstrate clarity of beliefs and values and knowing oneself. The care environment focuses on the context in which care is delivered and is contributing towards achieving PCC. The characteristics of the care environment are appropriate skill mix, systems that facilitate shared decision‐making, sharing of power, effective staff relationships, organizational systems that are supportive, potential for innovation and risk‐taking and the physical environment. The person‐centred processes focus on activities for delivering care and making the person‐centred practice work. The activities are working with patients’ beliefs and values, engaging authentically, being sympathetically present, sharing decision‐making and providing holistic care. The last domain, person‐centred outcomes, represents the anticipated results. The outcomes are a good care experience, involvement in care, a feeling of well‐being and the existence of a healthful culture (McCormack & McCance, 2016).

An international health policy survey in 2014 found that Sweden ranked at the bottom of 10 countries when it came to involving patients in their care and decision‐making (Commonwealth Fund, 2014). Different health regulations have been launched mandating that professionals practise according to a PCC approach. According to Swedish law (SFS, 2017:612), the region and the municipality shall collaborate and establish a collaborative plan for persons needing health care and social care. The law also transferred the responsibility for collaborative planning after hospital discharge to the health centres (HC), and the collaborative planning should preferably take place after the patient has arrived home (SFS, 2017:612). The new way of working changed the roles and responsibilities for the professionals involved in healthcare and social care organizations. Earlier studies in the north part of Sweden have indicated that the collaborative planning process is complex and the new way of working is challenging for the professionals (Jobe, Engström, & Lindberg, 2019; Jobe, Lindberg, Nordmark, & Engström, 2018).

We wanted to explore how the person‐centred practice framework developed by McCormack and McCance (2016) can add further knowledge to an area often described as multifaceted. Accordingly, this study aims to explore how the person‐centred practice framework can be applied to professionals participating in collaborative planning.

## 3 STUDY DESIGN

The study was conducted using an explorative, deductive approach (Elo & Kyngäs, 2008).

### 3.1 Method

Two domains of the person‐centred practice framework (McCormack & McCance, 2016), the care environment and person‐centred processes (Table 1), were analysed using deductive content analysis inspired by Elo and Kyngäs (2008). The data used did not contain attributes of the informants or results of the collaborative planning. Therefore, the other two domains of the framework, prerequisites and person‐centred outcomes, were omitted.

**TABLE 1:** Framework domains used for the analysis

Domain	Attributes
The care environment	Appropriate skill mix Shared decision‐making systems Effective staff relationships Power sharing The physical environment Supportive organizational systems Potential for innovation and risk‐taking
Person‐centred processes	Working with patients beliefs and values Sharing decision‐making Engaging authentically Being sympathetically present Providing holistic care

### 3.2 Participants

Eleven professionals, working for either the region or municipality (Table 2), from two different municipalities in the northern part of Sweden were asked to participate in the study. Purposive sampling was used to select participants as we sought to include a variety of professionals participating in collaborative planning (Sandelowski, 1995). Managers from two municipalities and three health clinics helped find participants and informed them about the study. The first author then contacted those 11 who had agreed to participate.

**TABLE 2:** Participants

Profession	Age	Sex	Working experience	Work place
Occupational therapist	59	Female	35	Municipality
Nurse	52	Female	28	Municipality
Social worker	38	Female	5	Municipality
Unit manager	45	Female	10	Municipality
Physiotherapist	43	Female	18	Municipality
Unit manager	35	Female	2	Municipality
General Practitioner	64	Female	38	Region Norrbotten
Occupational therapist	54	Female	30	Municipality
Nurse	46	Male	15	Region Norrbotten
Nurse	31	Female	3	Municipality
Nurse	38	Female	13	Region Norrbotten

### 3.3 Data collection

Data were collected between November 2018–January 2019. Semi‐structured interviews were used to collect data. Participants were asked about their experiences of working with the collaborative planning process and working according to a person‐centred approach. The participants were also presented with previous research findings related to patients and informal caregivers’ experiences of the collaborative planning conference and asked for their thoughts (Jobe et al., 2018, 2019).

### 3.4 Data analysis

The audiotaped interviews were transcribed and read through several times, to gain an initial impression of the content and to search for features and patterns. The framework for person‐centred practice (McCormack & McCance, 2016) served as the explanatory background guiding the interpretation, understanding and coding of the data material. Meaning units belonging to the two domains (the care environment and the person‐centred processes) of the framework were extracted and among them patterns and relationships were searched for. Meaning units sharing the same central meaning were grouped together under the corresponding attribute in the framework. A descriptive text conveying different aspects of the attributes was formulated (Elo & Kyngäs, 2008). The analysis process continued until no further abstraction was deemed appropriate.

### 3.5 Ethical considerations

All participants received verbal and written information about the study, and signed informed consent was collected. They were informed of their voluntary participation and right to withdraw at any time without further explanation and that their confidentiality would be guaranteed when the results were presented. The Ethical Regional Board, Umeå, Sweden, granted permission for the study under number dnr 2016/397‐31.

## 4 RESULTS

To present the findings, we used the attributes from two domains from the person‐centred practice framework, the care environment and the person‐centred processes (McCormack & McCance, 2016). The attributes are described below and illustrated with quotations.

### 4.1 The care environment

#### 4.1.1 Appropriate skill mix

The new way of working had created new roles and responsibilities for both actors, Region Norrbotten (responsible for the HCs and hospitals) and the municipalities, and the professionals when the collaborative planning had moved from the hospital to the patient's home after discharge. The HCs had not participated in the collaborative planning before, but with the new law they were now in charge and summoned the participants. The participants agreed that it was vital that the ones attending the conference were carefully selected, depending on the purpose of the conference, and had a mandate to make decisions. However, some HCs summoned all professionals available and expected the professionals themselves to decide if they should attend or not. Others selected participants based on the identified problems and needs of the patients.

*The one summoning the participants need to know or understand what kind of patient it is. You need to know what the problems and needs are, but you also need to know what the different professionals are doing and what their roles are… I can understand that it is sometimes difficult to know who is doing what…* (Nurse HC)

To facilitate decision‐making during the conference, professionals, within both health care and social care, delegated selected areas for decision‐making to other professionals if they themselves were not participating.

#### 4.1.2 Shared decision‐making systems

The actors had an overall agreement related to the collaborative planning process and worked according to a person‐centred approach. They had different systems for patients’ medical records. Professionals working at the municipality could access parts of the patient's medical record after informed consent from the patient. Professionals from health care and social care had not discussed and agreed together on a mutual definition of the person‐centred approach or a way to practice the collaborative planning process. Individual professionals also had different prerequisites, and interpreted and carried out person‐centred practice and the collaborative planning process in diverse ways. A majority of the communication between professionals was digital communication through a shared e‐platform between the actors. The e‐platform aided the professionals to follow the planning process. Through it, they could also see at which stage of the process the patient was and what parts of the planning other professionals had carried out or not carried out.

*With the new way of working, things have improved. I can follow the patient and be part of the whole process from the time he or she is admitted to the hospital until discharged, and I can see what others are planning and thinking. That, I think, is a benefit*. (Nurse, municipality)

Meetings between actors used to take place only at the management level, and the professionals wished there were regular opportunities for them too to meet, discuss, reflect and learn from each other.

#### 4.1.3 Effective staff relationships

The new way of working had improved the teamwork within the municipalities during the collaborative planning process, and they thought they had an excellent collaboration, both with fellow colleagues and also between professionals with different specialities. Their teamwork and communication were facilitated by physically sitting together as a team at the same place.

*We have to work together, and at the municipality we have a continuous dialogue. The rehab personnel and we in home health care (nurses, nurse assistants and managers) are using the same office*. (Unit manager, municipality)

Relations between the actors were more complex. Participants expressed discontent with actors and professionals who did not assume responsibility, did not do their part in the process and were hard to reach. There could also be conflicts between professionals related to what care and services they thought the patients should get.

#### 4.1.4 Power sharing

Participants described a smooth relationship with colleagues on different levels of the healthcare system. For instance, the occupational therapists or the physiotherapists at the municipality could easily contact and ask their colleagues at the hospital to carry out any assessment they wanted or thought was lacking before the patient went home. On the other hand, a hierarchy among areas and professionals also became evident. For example, medical staff felt that social care was prioritized in the patient's home and they were just seen as consultants. Participants from the municipality felt there were gaps in the process between actors when the patient was discharged. Not all HCs assumed responsibility or followed the process and participants from the municipalities thought the HCs wanted to transfer the responsibility of the patient to the municipality.

*The HCs want to hand over the patient and the responsibility to the municipality. You take over. It feels strange since they have the medical responsibility*. (Social worker, municipality)

There were times when professionals from the municipalities wished the HC did not accept the patient to be discharged from the hospital. Professionals from the two actors did not always agree on when to hold a conference or the definition of what a home healthcare patient entailed.

Those working at the HCs were frustrated over their new role of being in charge of the process and summoning the participants for the conference. They felt they had the least knowledge of the patient and depended on other professionals’ assessments to carry out their work and make their decisions.

*You do not want to be the stumbling block in the process, so you feel responsible to do your part… We have been given high authority with minimal information, so you have to trust what the professionals at the hospital have assessed. If it is written that the patient wants a conference and there are needs of further medical interventions, we will summon to a conference*. (Nurse HC)

#### 4.1.5 The physical environment

The majority of conferences took place in the patients’ home. The professionals thought it was very beneficial to meet the patient in their own environment. However, pressure from the management was exerted to use ICT solutions and they wanted the professionals to participate by video link from their offices during the conferences as it would save time and travel expenses. However, participants had experienced many technical problems with ICT and they did not feel it was optimal for the patients, many of whom were older and had cognitive disabilities.

*From the management, they want us to have more ICT‐based meetings, to use Skype. I am concerned. I do not feel it is working well with our old patients. There are few that can use it*. (GP, HC)

#### 4.1.6 Supportive organizational systems

The flow of patients had become faster, and severely ill patients were treated at home. The new way of working required a lot of time from the professionals, and no new resources had been added or tasks removed. They had to be available all the time, and it was difficult to plan other work ahead. This affected their working situation and environment. Participants felt the organizations were stuck in old structures, making it difficult to change the way of work and be flexible. Planning depended on individual professionals who were trying to follow the patient's goals and needs, and from an organizational perspective, the focus was still on deciding to which actor the patient belonged.

Due to high turnover of staff, the participants felt there was a need for continuous education in the planning process and e‐platform. To be successful, the professionals needed to be supported and acknowledged by the management.

*I think it has to do with how dedicated the managers are in the different working places. Our manager is very engaged and supportive and then we become committed. I believe it differs from place to place*. (Nurse, HC)

#### 4.1.7 Potential for innovation and risk‐taking

Guidelines had been developed for the collaborative planning process and conference. Nevertheless, not all professionals followed the guidelines or interpreted the tasks and the planning process in the same way. The many different ways of working caused frustration. At the same time, the participants did not want guiding principles that were too narrow.

*You cannot have strict guidelines. When they are not strict, there can be many different interpretations and if there are different interpretations, we just have to be better in follow‐up since we want the freedom to do things differently*. (Physiotherapist, municipality)

### 4.2 Person‐centred processes

#### 4.2.1 Working with patients’ beliefs and values

Before the conference, each professional assessed the needs of the patient. Not all of them contacted the patient and made the assessment together with the patient. Participants expressed concern for trusting the patient's views at times. They also felt they were not given the time and resources needed to make the assessment in a good way, and many times, they had to rely on informal caregivers and other professionals to provide the information needed.

*It is important to start with the individual it is the persons plan. I use to identify the persons needs and how we will … I ask what the persons own goals are? I assess for example how the rehabilitation is working. Can you walk without walker? Can you get out from bed yourself? What assistance and services will you need* (Social worker, municipality)

The professionals participating in the conference should document their agreement with the patient in the plan. There were workplaces that had discussed and agreed on how to write the documentation, while others left that to the individual professional.

#### 4.2.2 Sharing decision‐making

During the conference, the professionals should be setting goals together with the patient. Participants were frustrated with the quality of the conferences and felt that many of them did not serve their purpose. It felt more like an administration tool then an opportunity to agree on the care and services together with the patient.

*We have to change our way of working to a person‐centred approach. We have been working above the patient’s head before and done planning for me as a professional what I should do. But now it is the patients’ own planning and I need to take a step back and ask them questions in a different way and it is really difficult to make this adjustment*. (Occupational therapist, municipality)

Even if the conference took place in the patient's home, the participants felt the power balance was a difficult issue. Having so many professionals involved made it an unnatural situation and difficult for the patient to be the main partner. They would like to reduce the number of professionals to as few as possible.

#### 4.2.3 Engaging authentically

During the interviews, it became apparent that person‐centred practice and the view of the patient as a person did not mean the same thing to every professional. Not all of the professionals understood their role during the conference. Professionals that listened to and saw the patient and his or her needs formulated goals together and used their professional knowledge to break the goals down into objectives became frustrated working with colleagues practicing a different approach. There were also different ways of carrying out the conference. Commonly, the professionals took turns in discussing the identified issues and needs with the patient and participants felt they lacked a method for conducting mutual planning together with the patient, as a team, around goals acknowledged by the patient.

*A disadvantage has been that everyone is working in their corner. Everyone starts the planning process and so on. Then, when it is time for the conference, some feel they do not have to participate because they have already done such a huge part so they will not come. They do not understand the purpose and the patient has been told they will come and everyone will discuss together, so why does it become like this?* (Nurse, municipality)

#### 4.2.4 Being sympathetically present

The new way of working provided more opportunities for direct contact with the patient and the e‐platform made it easier to engage with the patient since the professionals could easily access information. However, not every professional managed to recognize what was unique with or important for the patients.

*I had a patient who was 97 and asked during the conference if she could get help to go to the bathhouse so she could go swimming. No one could answer her or find a solution. One even laughed at her question. If you are 97, they expect you to sit at home doing nothing. I wanted to take her in my car and drive her there, but I’m not allowed*. (Nurse Municipality)

#### 4.2.5 Providing holistic care

The role as a coordinator involved seeing and taking responsibility for the whole patient. Professionals found it challenging to give equal weight to all dimensions of the patient and not just to their own professional expertise. When a patient was discharged from the hospital, the municipality would provide a standardized package of care and services in the beginning until the level of care and services that was needed could be decided. With a minimum of resources and with time pressures, there was a risk that patients could continue to get services they were not in need of, thereby making them dependent. Overall, participants struggled with flexibility within the system and possibilities to see and cater to the unique patient and their capabilities.

*We should build on the patients’ capabilities and support them instead of offering services they are not in need of. For example, we never train or educate them. We run and provide. We never ask whether the patients have tried this themselves. No, it is much faster to say the person has a cognitive disability and needs help*. (Nurse, municipality)

## 5 DISCUSSION

The person‐centred practice framework presents attributes and relations between them for professional practice (McCormack & McCance, 2016). The framework analysis offers new insights into how PCC is expressed in practice during collaborative planning between the patient and healthcare and social care professionals from the professionals’ perspective.

Practice development and implementation of PCC and collaborative planning includes substantial organizational skills and attitudinal changes across health care and social care (Eaton et al., 2015) and commitment from organizations, management teams and professionals. The organization and management need to understand the culture and context in which they work and the characteristics that may prevent them from practising effectively (McCormack, Dewing, & McCance, 2011). Health and social care organizations are not designed to be integrated. They have different laws, budgets, geographical boundaries, IT systems, cultures and education of personnel (Glasby, 2016).

The results of our study disclosed that the healthcare and social care professionals were focused on enabling integrated care on an organizational and professional level, such as deciding to which actor the patient belonged. During this process, they made different interpretations. Professionals from different organizations attempt to keep the budgets of their organization balanced and therefore interpret issues differently in order to avoid responsibility for the costs (Dunér & Wolmesjö, 2015). According to Greenfield et al. (2014), integrated care has a macro and structured view and requires coordination of professional and organizational processes. PCC has a micro viewpoint and focuses on the interactional level between professionals and patients. The two concepts operate from different perspectives and the challenge for the management is to embed them together without one perspective either dominating or hindering the other.

The results revealed that professionals believed they were already practicing PCC. However, they lacked a common understanding of the concept and practice in relation to collaborative planning. PCC is more than a set of techniques, skills and procedures. It is a personal way of approaching, connecting and partnering with the patient (Edvardsson, 2015) and a specific culture that everyone in the organization needs to apply (Dewing & McCormack, 2017). The culture and a willingness to change may vary within and between organizations. Culture change requires a transformation of patterns and approaches. Creating a PCC culture is an ongoing commitment (McCormack & McCance, 2016). To embed PCC values in an organization is a process that takes time. It starts with agreeing on values and beliefs and then espousing the values, followed by living the values and finally having the structures and processes in place that are rooted with the values and beliefs to sustain them over time (McCormack, Manley, & Titchen, 2013).

Carlström and Ekman (2012) showed that a culture of human relations reduced change‐resistant and routine‐seeking behaviour and flat hierarchal structures. Furthermore, they found that social competences contributed to decreased tendencies to resist change to PCC. Practice development is a complex intervention and strategies facilitating it have been identified to be a shared purpose, including reflective feedback and evaluation processes (Manley, 2016). A shared purpose has also been shown to be a powerful strategy for unifying diverse organizations, enabling them to work together in the same direction and embrace agreed values (Manley, O'Keefe, Jackson, Pearce, & Smith, 2014). There are no shortcuts when implementing a new way of working. Organization and management need to take the time required to reach a shared understanding, purpose and joint way of practicing PCC and collaborative planning within their own organization and between organizations.

Lack of integration among professionals affected the PCC practice and the collaborative planning process. The participants did not plan together as a team and decisions made were profession‐specific. However, a decision made by one professional affected the decisions of the others in the team. These findings correspond with a study by Duner (2013) that also identified clarification of all professionals’ roles as vital in relation to care planning. The result of the study showed that professionals lacked knowledge of each other's roles, practiced domain thinking and struggled with power, trust and responsibility. According to Mangan, Miller, and Ward (2015), the lack of knowledge of other professionals’ roles and perceptions of different value bases can readily lead to stereotyping, poor communication and an unspoken hierarchy, thus preventing a change of culture.

For successful interprofessional collaboration, it is vital that all team members show strength in their own role and demonstrate knowledge of the other team members’ roles and also recognize their strengths in practice (Wei, Webb Corbett, Ray, & Wei, 2020). Interprofessional collaboration and shared decision‐making in care plan development are a complex phenomenon. A study by van Dongen et al. (2016) identified five categories of factors influencing the process. They were patient‐related factors (e.g. active role of the patient, formulation/language of patients'goals and wishes), professional‐related factors (e.g. individual competences, domain thinking), interpersonal factors related to the interaction between team members (e.g. use of discipline‐specific language, trust and respect), organizational factors (e.g. shared vision and mission, leadership) and external factors (e.g. law and regulations, finance). Teams can work in different ways and function differently during various circumstances. Working in interprofessional collaboration means that each professional brings his or her own unique skills and expertise to the team and in an interpersonal process, together with the patient, attains goals that could not be achieved by one team member alone (Jones & Phillips, 2016).

The participants in the study struggled with seeing the person behind the patient, identifying the person's needs and offering services and interventions tailored for the person. Changing this way of working is difficult (Carlström & Olsson, 2014) and requires a new way of thinking, delivering services and building relationships. A completely new system approach is needed (Eaton et al., 2015). Using standard protocols (e.g. the Comprehensive Geriatric Assessment) helps to include all aspects of the person and achieve a holistic approach in the collaborative care plan (Phillips, Mcmillan, Gibb, & Reed, 2017). The PCC centre in Gothenburg has developed a PCC health plan at discharge that includes the patient's narrative, resources, motivations and goals. The social situation at home and the activity level of the patient is also considered. The plan created covers the care and the gap between hospital and home (Ulin et al., 2016). A study by Wolf et al. (2017) pointed out that PCC made patients feel safe and secure and increased their confidence in professionals. Patients appeared to value a human connectedness above formalized aspects of documenting agreed goals and care planning. The management must not only provide antecedents, vision and commitment to PCC but also a change in attitudes and behaviours. They have to convey and value understanding of what it means to be with and care for a person, rather than focusing solely on doing something for the person (Washburn & Grossman, 2017).

Leadership is well known to have a strong influence on organization and workplace cultures (Cardiff, 2016). Professionals have a responsibility to reflect on their own practice and relationships and participate in giving and receiving feedback to assist in building effective relationships, teams and workplaces (Manley, 2016). However, if the professionals do not experience person‐centeredness themselves, it will be difficult for them to work in a person‐centred way (McCormack & McCance, 2016). Person‐centred leadership focuses on well‐being and empowerment but also takes into consideration the context. It enables the leader to be in relation with the other team members and to facilitate workplace learning (Cardiff, 2016). As a participant in our study said, an engaged leader creates engaged professionals. WHO (2015) proposes distributed leadership between actors across professional and organizational boundaries in order to achieve people‐centred and integrated health services. Co‐leadership and co‐location of services create a perception of the management role as a collective activity (Klinga, Hansson, Hasson, & Sachs, 2016). The professionals in the study stationed at the same offices in the municipality expressed satisfaction with their communication and collaboration.

In Sweden, as in many other countries, education and training are usually offered separately for each profession (Hägg‐Martinell, Hult, Henriksson, & Kiessling, 2019). If students and professionals learn from, with and about each other during education and training, they will be better prepared to deliver integrated and person‐centred care during practice (Machin et al., 2019). There is a need to adapt the education and training of healthcare and social care professionals to correspond with integrated and person‐centred practice (Nolte, 2017).

## 6 STUDY LIMITATIONS

This study has limitations; for example, only 11 participants were interviewed. When determining the sample size, we judged the quality of the data collected against the specific aim. Variation was required to allow deep analysis of the data, and the participants described experiences that were rich in content, which revealed a pattern that we found served adequately as a basis for the findings (Sandelowski, 1995).

## 7 CONCLUSION

Using the person‐centred practice framework in the analysis of the data offered new insights into the PCC aspects of the collaborative planning process and highlighted the need for a systemic approach when implementing PCC. Professionals need to understand the values required to practice PCC and the difference between being with and caring for a person compared to only doing something for a person. There is a need for more research using the person‐centred practice framework within different contexts to facilitate learning from and about good (and insufficient) examples of person‐centred practice to increase the body of evidence.

## CONFLICT OF INTEREST

We declare there is no conflict of interest.

## AUTHOR CONTRIBUTIONS

IJ, ÅE and BL designed the study, analysed the data and wrote the manuscript. IJ collected the data.

## Data Availability

The original data are in Swedish language. Due to the promise of confidential presentation of the participants, we prefer not to share these data.
